# A metabolomics approach to target the effect of histone protein methyltransferase-specific probe UNC1999 on cryptic metabolites in an endophytic fungus *Xylaria papulis* Lloyd

**DOI:** 10.3389/fmicb.2026.1894461

**Published:** 2026-08-14

**Authors:** Shubham Vishwakarma, Jay Hind Nishad, Anjali, Shagun Sinha, Rajnish Bharti, Laxmi Shekhar, Ravindra Nath Kharwar

**Affiliations:** 1 Department of Botany, Institute of Science, Banaras Hindu University, Varanasi, India; 2 Plant Tissue Culture and Genetic Engineering Lab, Department of Biotechnology, National Agri-Food and Biomanufacturing Institute (NABI), Ministry of Science and Technology (Government of India), Mohali, Punjab, India; 3 Department of Botany, D.A.V.P.G. College, Azamgarh, India

**Keywords:** cryptic metabolites, endophytes, histone methyl transferase inhibitors, microbial epigenetics, natural compounds

## Abstract

*Xylaria papulis* Lloyd, an endophytic fungus, was isolated from the stem of *Andrographis paniculata* Willd., commonly known as green chiretta, which is native to India and Sri Lanka. To date, no report is available on the epigenetic modulation of *X. papulis* using the histone methyltransferase inhibitor UNC1999. Hence, this study aims to evaluate the independent effect of this probe. The fungal culture was evaluated for antioxidant (2,2-diphenyl-1-picrylhydrazyl and 2,2′-azino-bis(3-ethylbenzothiazoline-6-sulfonic acid radical scavenging assays) and antibacterial potential against six human pathogens: *Staphylococcus epidermidis* (ATCC 12223), methicillin-susceptible *Staphylococcus aureus* (MSSA) (ATCC 25923), methicillin-resistant *Staphylococcus aureus* (MRSA) (MU50), *Enterobacter cloacae* (ATCC 13047), *Klebsiella pneumoniae* (ATCC 700603), and *E. coli* (ATCC 25922). The crude extract, which was recovered from the samples treated with 250 nM of UNC1999, exhibited enhanced antioxidant and antibacterial efficacy against *S. epidermidis* (ATCC 12223), MSSA (ATCC 25923), MRSA (MU50), *E. cloacae* (ATCC 13047), and *K. pneumoniae* (ATCC 700603), compared to the control. The chemical profiles of crude extracts, both treated and untreated, were analyzed using gas chromatography–mass spectrometry and ultra-high-performance liquid chromatography along with high-resolution mass spectrometry). The results highlighted the capacity of UNC1999 to modulate the synthesis of cryptic metabolites in *X. papulis*, displaying antimicrobial and anticancer potential. This study underlines the importance of further research into the molecular mechanisms regulating these epigenetically modulated metabolic pathways.

## Highlights

- A fungal endophyte (APL1) was obtained from the *Andrographis paniculata* leaves and subsequently identified as *Xylaria papulis*.
- Histone methyltransferase (HMT) inhibition using UNC1999 effectively activated cryptic biosynthetic pathways in *X. papulis.*
- Metabolomic analysis revealed significant alterations in the profile of secondary metabolites profile upon epigenetic modulation.
- Induction of previously silent gene clusters can result in the biosynthesis of novel and structurally diverse metabolites.
- Comparative metabolomic profiling demonstrated the enhanced chemical diversity in treated fungal cultures versus control.
- UNC1999 treatment underscores the potential of epigenetic probes as tools for uncovering natural products from endophytic fungi.
- This study reveals the chromatin-level control of secondary metabolic pathways in *X. papulis*.
- The findings highlight the applicability of metabolomics-driven strategies for uncovering hidden fungal metabolomes.

## 1 Introduction

The term “endophyte” refers to any microorganism, which can be actinomycetes, bacteria, or fungi, that inhabits the tissues of a healthy plant without eliciting discernible disease symptoms in the host. Utilizing endophytes as an alternative reservoir for plant-derived drugs, such as camptothecin, vincristine, vinblastine, rohitukine, azadirachtin, and piperine, holds the potential to alleviate both cost-related challenges and the ecological crisis associated with host-mimetic compounds (Deshmukh et al., 2019). This approach offers a strategy for price reduction and addresses the growing concern of the overexploitation of host plants by providing a sustainable alternative source of valuable compounds without depleting natural resources. *Xylaria papulis*, a member of Ascomycota, belongs to the class Sordariomycetes and belongs to a large family, Xylariaceae (Kim et al., 2025). There are reports that describe the structurally distinct polyketides derived from *X. papulis*. These include xylapapusides A and B, which lead to the inhibition of NO production in LPS-induced RAW264 (Becker and Stadler, 2021). Xylapapuside A exhibits the most potent NO inhibition, achieving an *E*max value of 34.3 μM. These compounds were reported from the cultures of an endophyte *X. papulis*, which had been isolated from the plant *Lepidagathis stenophylla* (Baraban et al., 2013).

Endophytic fungi produce numerous structurally varied and physiologically active secondary metabolites in large quantities, which have intriguing medicinal qualities. However, a significant proportion of these biosynthetic gene clusters (BGCs) is either weakly expressed or transcriptionally silent under typical laboratory conditions, which results in an underestimation of the actual biosynthetic capacity of these organisms (Pfannenstiel and Keller, 2019; Munusamy et al., 2022). Recent developments in epigenetic modifications have shown a promising approach to unlocking new metabolites and activating cryptic BGCs. Histone deacetylase and histone methyltransferase inhibitors are two examples of chemical epigenetic regulators that have attracted significant interest (Chiang et al., 2011; Zhang et al., 2020). One important epigenetic marker that is essential to chromatin remodeling and the control of gene expression is histone methylation. Transcriptional repression is linked to the methylation of lysine residues on histone H3, specifically H3K27 (Nichol et al., 2016). HMT inhibitors have been effectively used to change the chromatin state and activate dormant genes by inhibiting methylation of histone protein and making chromatin transcribable. A strong and selective inhibitor of EZH2 and EZH1, which are members of the polycomb repressive complex 2 (PRC2) that methylates H3K27, is UNC1999 (Konze et al., 2013; Xu et al., 2015). UNC-1999 was first created for cancer treatment; however, microbial epigenetic research has shown considerable potential in triggering the expression of otherwise silent (cryptic) and unexplored secondary metabolites.

This research examines the impact of UNC-1999 on the production of secondary metabolites in the endophytic fungus *X. papulis*, a species renowned for its wide range of biosynthetic capabilities using a metabolomics-based methodology. The study analyzed the overall metabolic changes brought on by UNC1999 treatment to find novel or differently expressed metabolites that might indicate activated cryptic pathways by combining untargeted metabolomics with multivariate statistical analysis. In addition to improving our understanding of the epigenetic control of fungal metabolism, this method facilitates the discovery of unexplored bioactive natural compounds that may have medicinal applications.

## 2 Materials and methods

### 2.1 Fungal endophyte material

*Xylaria papulis* (APL1), a fungal endophyte, was isolated from mature, healthy leaves of *Andrographis paniculata* obtained from the Botanical Garden of FRI, Dehradun, India. The plant material was randomly sampled, placed in sterilized polybags, and kept in an icebox at 4 °C prior to subsequent laboratory analysis. According to Nishad et al. (2025), for the surface sterilization, plant samples were sliced into 5 × 5 mm^2^ segments, then sequentially submerged in ethanol (70% EtOH) for 45 s, followed by 2% NaOCl (sodium hypochlorite) for 30 s and again in 70% ethanol for 45 s. Subsequently, the segments underwent a complete immersion in sterile distilled water to remove ethanol and NaOCl. Plant tissue segments were air-dried and placed onto antibiotic-supplemented potato dextrose agar (PDA) plates. Sealed with laboratory parafilm, the plates were incubated in a biological oxygen demand incubator at 28 ± 2 °C for 5–7 days. After colonies of fungus appeared on the PDA plates, the fungal endophyte was isolated on a fresh plate and purified.

### 2.2 DNA extraction and PCR amplification

The mycelium of *X. papulis* (APL1) was grown aseptically in potato dextrose broth (PDB) media and maintained at 28 ± 2 °C for 7 days. The fungal mycelial mat was aseptically harvested and subsequently utilized for DNA extraction. DNA was extracted using the cetyltrimethylammonium bromide method described by Murray and Thompson (1980) with slight modifications. The genomic DNA concentration was assessed via the Thermo Scientific UV–Vis spectrophotometer NanoDrop^™^. Furthermore, PCR amplification was performed targeting the internal transcribed spacer region (ITS) (White et al., 1990) and actin (ACT) (Carbone and Kohn, 1999). The amplified products were separated by electrophoresis on a 2.0% (w/v) agarose gel and visualized following staining with ethidium bromide (Sinha et al., 2025). A DNA mini kit was used to purify the amplified DNA bands via gel excision before nucleotide sequencing using the Sanger method by EDZ Daignon Pvt., Ltd., India.

### 2.3 Phylogenetic analysis

**Figure 1:**
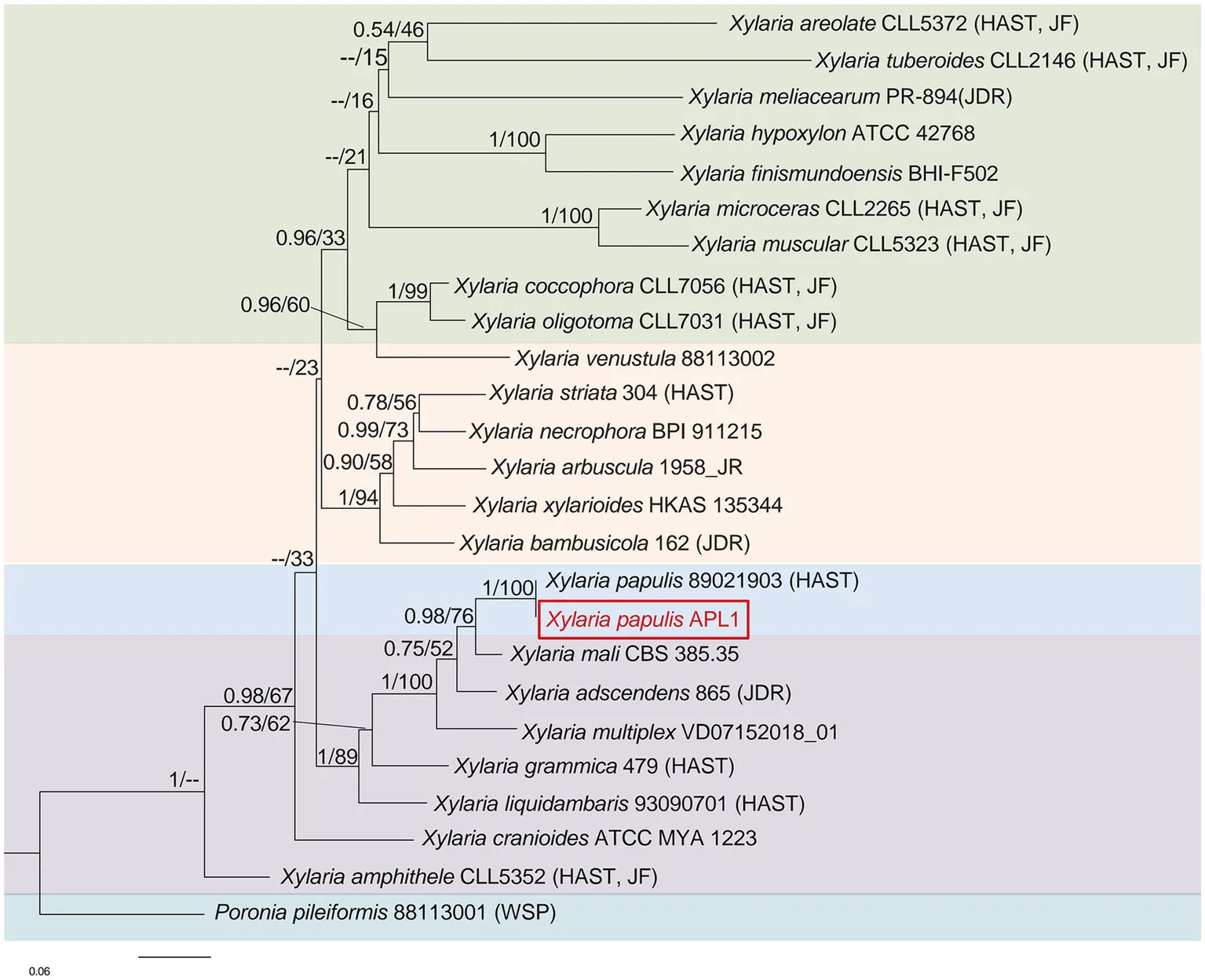
A maximum-likelihood (ML) tree based on the concatenated sequence data of ITS and ACT showing phylogenetic affinities of sequenced *Xylaria papulis* (AP1) with other species. Bayesian posterior probabilities (PP) and ML bootstrap support (BS) values are shown at the nodes as PP/BS, respectively. ML bootstrap support (BS) ≥ 75% and Bayesian posterior probabilities (PP) ≥ 0.90 are given at the nodes in this order. The tree was rooted to *Poronia pileiformis*. The scale bar represents 0.06 substitutions per site.

The resulting sequences were assessed through quality-check using Chromas v.2.6.6 (Technelysium Pty Ltd). The concatenated ITS-ACT dataset was used for the construction of a phylogenetic tree. Using the NCBI BLAST search, reference sequences of closely related strains were obtained from GenBank (Supplementary Table 1). The phylogenetic trees were constructed using Bayesian inference (BI) in MrBayes v.3.2.7 (Ronquist et al., 2012), and maximum likelihood (ML) analysis in RAxML v.8.2.10 (Stamatakis, 2014; Edler et al., 2021). *Poronia pileiformis* (88113001) was used as an outgroup. Tree reconstruction, visualization, and editing were performed using FigTree v.1.4.4 (Rambaut, 2018), and the final layouts were prepared in Adobe^®^ Illustrator CC 2017. The produced multigene phylogram is illustrated in Figure 1.

### 2.4 Preparation of the UNC1999 solution for epigenetic treatment

An amount of 2.84 mg of UNC1999 (100 μM) was dissolved in 100 mL of dimethyl sulfoxide (DMSO) to prepare a stock solution. Afterward, to remove any potential contaminants, the sample was passed through a 0.20 μm syringe filter. To evaluate the effects of UNC1999, the appropriate volumes of the stock solutions were added separately to 100 mL of PDB medium to achieve concentrations of 25, 50, 100, 250, 500, and 1,000 nM. The endophytic fungal isolate was added to each treatment separately, and the treated samples were then cultivated at 27 ± 2 °C for 21 days. Each treatment was performed three times to ensure reproducibility.

### 2.5 Extraction of secondary metabolites

Endophytic fungal cultures were cultivated in 100 mL of PDB medium at 27 ± 2 °C for 21 days. After 21 days of incubation, the cultured broth was separated via sterile filter paper (Whatman No. 1) for isolating the fungal biomass and the culture supernatant separately. In a separating funnel, the supernatant underwent extraction with an equivalent amount of ethyl acetate (three times) to produce the crude extract. A rotary evaporator (IKA RV 10, Germany) was used for concentrating the combined organic layer. After weighing, the dried extract was mixed with a small amount of methanol (1 mg/10 μL) and kept at 4 °C for further examination.

### 2.6 Antibacterial activity

The fungal metabolites were obtained from various treatments of epigenetic modulators (UNC1999). Antibacterial potential was studied by the disc diffusion method against selected human pathogenic bacteria such as *Staphylococcus epidermidis* ATCC12223, methicillin-susceptible *Staphylococcus aureus* (MSSA) ATCC25923, *Klebsiella pneumoniae* ATCC700603, *Enterobacter cloacae* ATCC13047, and methicillin-resistant *Staphylococcus aureus* (MRSA) MU50. First, on the Mueller Hinton agar (MHA) plate, a lawn of bacteria was dispersed using cotton swabs. Then, sterile filter paper discs impregnated with different treatments of crude metabolites were placed on the test bacterial colony, while a separate filter paper disc was dried after loading with methanol (10 μL) as the positive control.

Every test was performed in triplicate. The antibacterial activity was assessed by measuring the zones of growth inhibition on plates that were incubated at 35 ± 2 °C for 24 h. The standard deviations and mean diameters of the inhibitory zones were estimated.

### 2.7 *In vitro* antioxidant activity

#### 2.7.1 2,2-diphenyl-1-picrylhydrazyl (DPPH) methods

The antioxidant activity of the isolated compound was evaluated based on the method described by Gautam et al. (2024), with slight modifications. One milliliter of 0.0079% DPPH (2,2-diphenyl-1-picrylhydrazyl) was added to methanol for each test tube containing 3 mL of fungal crude extract at concentrations ranging from 12.5 to 400 μg and kept for 15–20 min in the dark at room temperature. A reaction mixture containing only methanol and DPPH, excluding crude extract served as the control. After incubation, the O.D. values were recorded at 517 nm, using ultraviolet–visible spectroscopy (U-2900, Hitachi) against methanol as blank. For comparison of antioxidant activity, ascorbic acid was used as a positive control. The dose-dependent curve was used to calculate the free radical inhibiting percentage and EC_50_ value, which is the concentration required to eliminate 50% of DPPH radicals. Each test was run in triplicate, and the mean ± standard deviation was used to elucidate the results.


(Abscontrol–Abstest sample)÷Abscontrol×100

Where Abs test sample denotes the absorbance (O.D.) of the tested sample or standard, and Abs control denotes the absorbance of the control reaction.

#### 2.7.2 ABTS^+^ methods

The antioxidant potential using the ABTS^+^ radical scavenging assay was carried out as mentioned by Gautam et al. (2024), with slight modifications. After preparing ABTS^+^ radicals with equal quantities of 7 mM ABTS^+^ solution and 2.45 mM potassium persulfate, the mixture was stored at room temperature in the absence of light for 20 min. Following this, the ethanol was used to dilute the mixture until it reached an absorbance of 0.70 ± 0.05 at 734 nm. Two milliliters of ethanol and 1 mL of ABTS^+^ were mixed with a 20 μL aliquot of the sample at different concentrations (12.5–400 μg). A UV–visible spectrophotometer was used to measure the optical density at 734 nm. Ascorbic acid (AA) was used as a positive control. EC₅₀ was defined as the concentration required to eliminating 50% of ABTS^+^ radicals.


$$\%\text{Inhibition}=[({\mathrm{Abs}}_{\text{control}}-{\mathrm{Abs}}_{\text{test sample}})∕{\mathrm{Abs}}_{\text{control}}\times 100]$$

Abs _Control_ and Abs _Sample_ represented the absorbances (O.D.) of the ABTS^+^ in the control solution and sample, respectively. Antioxidant activity in the sample was estimated by plotting the calibration curves against different concentrations of both sample and reference solutions. Each test was conducted in triplicate.

### 2.8 GC–MS analysis

Using a TSQ Duo device (Thermo Scientific) fitted with a TG-5MS capillary column, the fungal crude metabolites were identified using GC–MS. The analysis was conducted at the Department of Botany, Banaras Hindu University (BHU), Varanasi, Uttar Pradesh, India. The temperature of the GC oven was first kept at 70 °C for 3 min, after which it was designed to ramp up to 300 °C at a rate of 8 °C/min. As a carrier gas, helium was used at a constant flow rate of 1.0 mL/min, while the injector temperature was kept at 250 °C. The analytes were ionized using the electron impact (EI) mode at 70 eV. Mass spectra were obtained throughout a scanning range of 40–450 *m*/*z* after crude metabolite samples were injected in split mode with a 40:1 ratio. By comparing the obtained mass spectra with reference information from the Wiley 275 and NIST, USA spectral libraries, bioactive components were found. The compounds’ names, molecular weights, retention times, and documented biological activities were recorded.

### 2.9 Ultra-high-performance liquid chromatography (UHPLC)-Q-TOF-MS/MS analysis

An Orbitrap Eclipse Tribrid Mass Spectrometer (Thermo Fisher Scientific, United States) was used to perform ultra-high-performance liquid chromatography along with high-resolution mass spectrometry (UHPLC-HRMS) analysis at SATHI-BHU, Banaras Hindu University (BHU), Varanasi, Uttar Pradesh, India. Small molecule chromatographic resolution was carried out using Compound Discoverer software (version 3.2.0.421) as well as using UHPLC-HRMS. A ternary mobile phase solvent system was employed with 100% water and 0.1% formic acid in solvent A, 80% acetonitrile and 0.1% formic acid in solvent B, and 100% methanol and 0.1% formic acid in solvent C. Metabolites were separated using a Hypersil GOLDTM C18 Selectivity HPLC Column (2.1 mm internal diameter × 100 mm length, 1.9 μm particle size). With a sample volume of 5 μL, a mobile phase delivery rate of 0.3 mL/min, and a total analysis time of 30 min were employed. A heated electrospray ionization (H-ESI) interface that functions in both positive and negative ion modes was used to route the column eluent into the mass detection device. High-resolution mass spectra were obtained using the Orbitrap analyzer. To identify the metabolites, the gathered mass spectral data were searched against many reference databases, including Predicted Compositions, mzCloud, ChemSpider, and MassList Search.

### 2.10 Statistical analysis

One-way ANOVA in GraphPad Prism software (Version 8, United States) was used for statistical analysis. Tukey’s HSD, *F*-statistics, paired *t*-test, and the mean ± SD of three independent experiments were used to present the results.

## 3 Results

### 3.1 Morpho-molecular identification

Based on microscopic characteristics and molecular analyses, the isolate was identified as *X. papulis*. The GenBank accession numbers assigned were ON796531 (ITS) and PX246287 (ACT). The phylogenetic relationships within the genus *Xylaria* were inferred using a concatenated dataset of ITS and ACT gene sequences. The maximum likelihood analysis revealed that *X. papulis* (APL1) is clustered within a well-supported clade of closely related *Xylaria* species. The isolate APL1 showed the highest sequence similarity and phylogenetic affinity with *X. papulis* 89021903 (HAST), forming a distinct subclade supported by strong bootstrap values (≥100%). APL1 evolutionary position within the genus is indicated by the robustness of this grouping. With ACT offering further assistance for deeper nodes and ITS helping with species-level discrimination, the combined ITS-ACT dataset improved the resolution of both intra-specific and inter-specific interactions. The observed genetic divergence between APL1 and its nearest phylogenetic neighbors suggests that it represents a distinct lineage within the *Xylaria* clade. This *X. papulis* clade is further grouped with closely related species such as *Xylaria mali*, *X. adscendens*, *X. multiplex*, *X. grammica* and *X. liquidambaris* with intermediate bootstrap values (≥ 52–90%). This suggests that these species share a common ancestor and belong to a closely related evolutionary cluster within the genus. The outgroup *Poronia pileiformis* 88113001 (WPS) formed a distinct clade (Figure 1).

### 3.2 Antibacterial activity of both treated and untreated cultures

**Figure 2:**
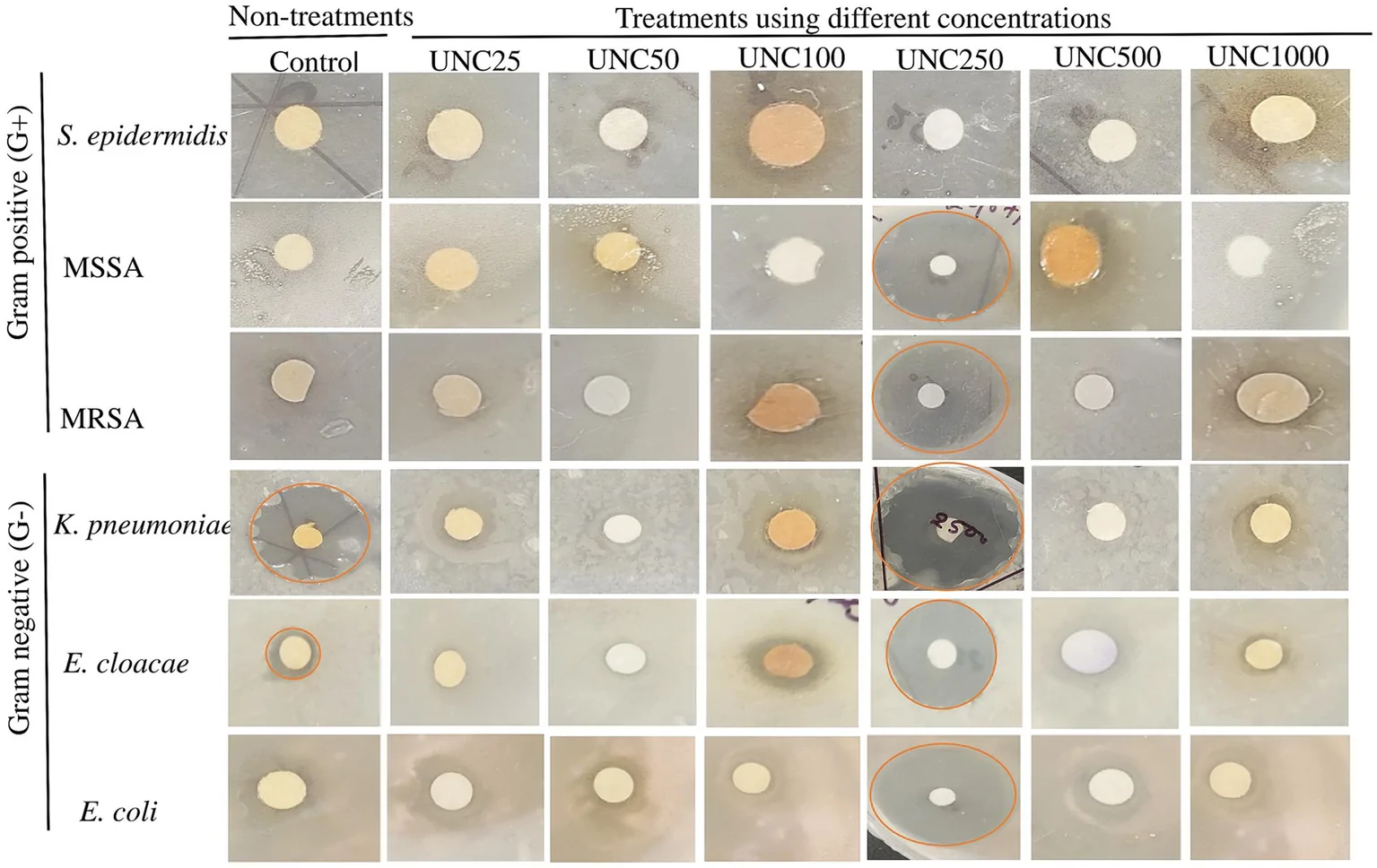
Antibacterial activity of crude metabolites from UNC1999 treated and non-treated (control) cultures of *X. papulis* against both Gram-positive and Gram-negative bacteria, assessed using the disc diffusion assay. Red circles indicate the zones of inhibition produced by the metabolites, compared with the control treatment.

The crude extracts obtained from the 250 nM UNC1999-treated cultures demonstrated significantly greater antibacterial activity by producing the inhibition zones of 28.13 ± 2.29 mm against *K. pneumoniae* (ATCC 700603), 29.06 ± 2.10 mm against MSSA (ATCC25923), 21.02 ± 1.75 mm against *E. cloacae* (ATCC13047), 25.10 ± 1.81 mm against MRSA (MU50), and 33.13 ± 1.79 mm against *E. coli*, but no zone of inhibition was found for *S. epidermidis* (ATCC12223) compared to that of the extract of the untreated culture (Figure 2). In contrast, lower and higher concentrations compared to 250 nM did not show a significant inhibitory effect against the bacterial pathogens tested, either in treated or untreated extracts (Table 1).

**Table 1:** Results of one-way ANOVA and Tukey’s HSD mean comparison examining the zone of inhibition against different human pathogenic bacteria using metabolites extracted from treated and non-treated (control) fungus *X. papulis*.

Human pathogenic bacteria	Control	25 nM	50 nM	100 nM	250 nM	500 nM	1,000 nM	*F*-statistics	*p*-value	Tukey’s HSD mean comparison
*Staphylococcus epidermidis* (ATCC12223)	NA	NA	NA	NA	NA	NA	NA	NA	NA	NA
*Klebsiella pneumoniae* (ATCC700603)	21.10 ± 1.25	11.11 ± 0.95	10.13 ± 0.92	13.03 ± 1.29	28.13 ± 2.29	NA	10.13 ± 0.76	24326.9	<0.0001	250 nM > control> 100 nM > 25 nM > 50 nM, 1,000 nM, 500 nM
MSSA (ATCC25923)	10.13 ± 0.58	NA	10.13 ± 0.92	9.30 ± 1.43	29.06 ± 2.10	NA	NA	11331.7	<0.0001	250 nM > control> 100 nM > 25 nM,50 nM, 1,000 nM, 500 nM
*Enterobacter cloacae* (ATCC13047)	9.10 ± 0.74	NA	NA	9.09 ± 0.58	21.02 ± 1.75	9.00 ± 0.56	9.03 ± 0.61	65483.1	<0.0001	250 nM > control> 500 nM > 100 nM > 1,000 nM, 25 nM, 50 nM
MRSA(MU50)	6.08 ± 0.68	6.09 ± 0.54	NA	6.05 ± 0.49	25.10 ± 1.81	NA	5.92 ± 0.62	42515.7	<0.0001	250 nM > control> 25 nM > 100 nM > 1,000 nM, 50 nM, 500 nM
*E. coli*	10.03 ± 1.05	NA	10.01 ± 0.90	9.95 ± 0.82	33.13 ± 1.79	10.02 ± 0.39	9.69 ± 0.59	91061.7	<0.0001	250 nM > control>500 nM > 50 nM > 100 nM > 1,000 nM > 25 nM

Data are presented as mean ± SD (*n* = 3). The 250 nM concentration showed a statistically significant difference compared with the control for all susceptible bacterial strains (***p < 0*.001*). Where NA represents no activity detected.

### 3.3 DPPH method

**Figure 3:**
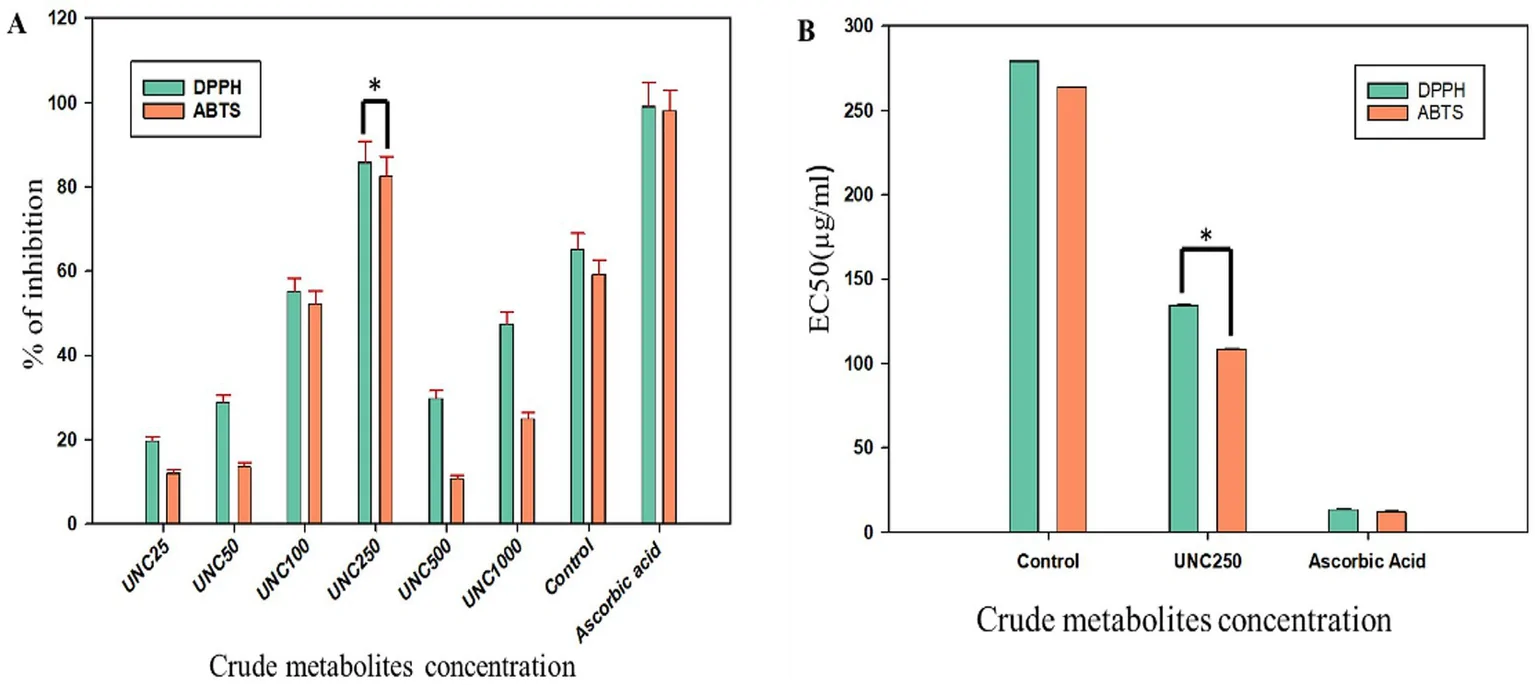
Antioxidant activity of crude metabolites from UNC1999-treated and non-treated (control) cultures. **(A)** DPPH free radical scavenging activity and ABTS•^+^ radical scavenging activity. **(B)** Comparison of EC_50_ values of treated and non-treated crude metabolites. Data are presented as mean ± SD. An asterisk (*) indicates a statistically significant difference compared with the control and other UNC1999 concentrations (*p* < 0.05).

The culture filtrate of *X. papulis* (APL1) treated with UNC1999 at 250 nM concentration, when tested for its antioxidant potential using DPPH, indicated a substantial free radical quenching ability compared to that of control and other treatments. The highest activity and EC50 of free radical scavenging were observed in UNC1999 at a 134.48 μg/mL concentration; an enhanced free radical scavenging activity trend was detected while the concentration of UNC1999 increased. UNC1999 of 250 nM indicated 82.49% scavenging activity, whereas the control showed 59.22% inhibition. DPPH is a persistent free radical that exhibits a deep violet color, which becomes pale yellow or colorless when reduced by an antioxidant. The antioxidant chemical may scavenge DPPH by donating a hydrogen atom to generate a stable DPPH-H molecule with an inability to absorb at 517 nm (Figure 3A). The drop in absorbance represents the sample’s scavenging potential. The control revealed a high EC_50_ value of 278.93 ± 0.48 μg/mL, indicating low antioxidant activity (Figure 3B). UNC1999 (250 μg/mL) treated fungal extract resulted in a large drop in EC_50_ to 134.48 ± 0.41 μg/mL, demonstrating a significant boost in radical scavenging activity. The standard, ascorbic acid, gave the lowest EC_50_ value (13.12 ± 0.254 μg/mL), showing its significant antioxidant capability (Table 2).

**Table 2:** DPPH % inhibition of 250 nM UNC1999 treated and non-treated (control) crude metabolites.

Metabolite concentration (mg/3 mL)	DPPH % inhibition		*t*-value	*p*-value
	Control (mean± SD)	UNC1999 treated (mean± SD)		
12.5	4.00 ± 0.065	25.55 ± 0.417	88.44	<0.0001
25	12.49 ± 0.204	36.46 ± 0.595	66.01	<0.0001
50	27.36 ± 0.447	40.18 ± 0.656	27.97	<0.0001
100	38.89 ± 0.635	54.37 ± 0.888	24.56	<0.0001
200	48.03 ± 0.784	62.45 ± 1.020	19.41	<0.0001
400	58.69 ± 0.958	79.04 ± 1.290	21.94	<0.0001

Data are presented as mean ± SD (*n* = 3). All treated groups showed significantly higher DPPH radical scavenging activity than the corresponding controls (*p* < 0.0001).

### 3.4 ABTS^+^ method

Additionally, the ABTS^+^ assay was used to evaluate antioxidant potential. ABTS^+^ radical cations (ABTS^+^), generated by reacting ABTS^+^ with potassium persulfate, exhibit a blue-green color with a strong absorbance at 734 nm. Antioxidants present in the sample neutralize the ABTS^+^ radicals, resulting in a measurable decrease in absorbance, which is indicative of the antioxidant capacity. Due to the very low redox potential of ABTS^+^, a wide range of phenolic compounds interact, and the ABTS^+^ could be useful in evaluating the antioxidant for hydrophilic and hydrophobic compounds due to its nature to dissolve in both inorganic and organic solvents. Similar to the DPPH assay, the free radical scavenging potential EC_50_ was observed in UNC1999 at a 134.48 μg/mL concentration; after this, there was a decline in free radical scavenging activity when the concentration of UNC1999 was increased (Figure 3A). UNC1999 of 250 nM demonstrated 85.77% scavenging activity, while the control showed 65.2% inhibition. The control showed an EC_50_ of 263.53 ± 0.29, whereas UNC1999 treatment reduced the EC_50_ to 108.40 ± 0.377, indicating improved antioxidant efficiency (Figure 3B). Ascorbic acid again exhibited the highest activity with the lowest EC_50_ value (12.17 ± 0.22) (Table 3; Supplementary Figure 1).

**Table 3:** ABTS^+^% inhibition of 250 nM UNC1999 treated and non-treated (control) crude metabolite.

Crude metabolite concentration (mg/3 ml)	ABTS^+^% inhibition		*t*-value	*p*-value
	Control (mean± SD)	UNC1999 treated (mean± SD)		
12.5	6.00 ± 0.008	25.38 ± 0.414	81.06	<0.0001
25	14.49 ± 0.237	38.63 ± 0.630	61.87	<0.0001
50	29.36 ± 0.479	43.81 ± 0.715	29.22	<0.0001
100	40.89 ± 0.668	49.84 ± 0.814	14.67	<0.0001
200	50.03 ± 0.817	74.14 ± 1.210	28.7	<0.0001
400	60.69 ± 0.991	82.87 ± 1.353	22.95	<0.0001

Data are presented as mean ± SD (*n* = 3). All treated groups showed significantly higher ABTS^+^ activity than the corresponding controls (*p* < 0.0001).

### 3.5 Characterization of bioactive compounds

**Figure 4:**
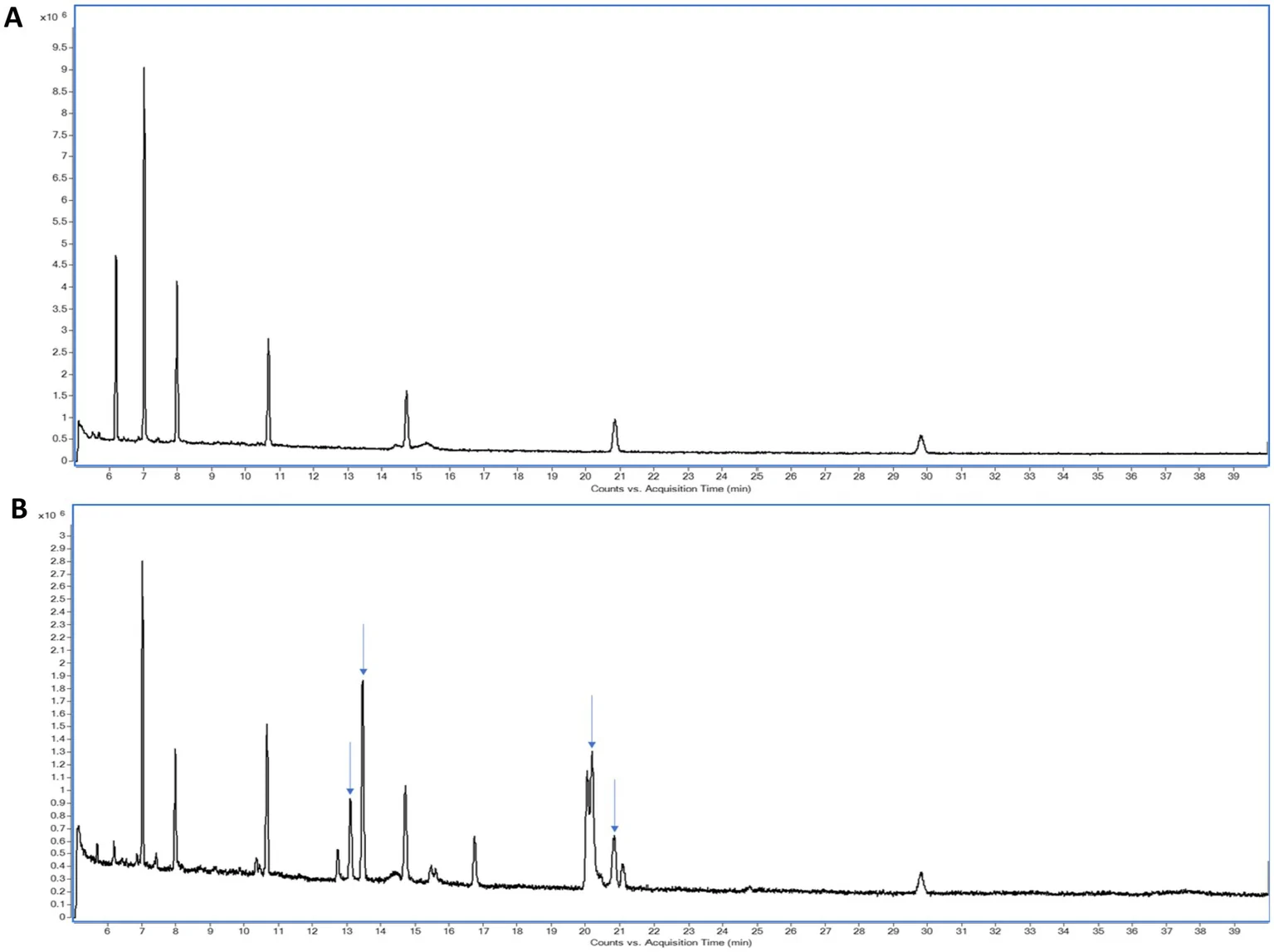
GC–MS chromatograms of crude metabolites from **(A)** the untreated (control) sample and **(B)** the 250 nM UNC1999-treated sample.

Secondary metabolite production was induced, according to GC–MS analysis of crude ethyl acetate extracts of cultures treated with 250 nM UNC1999 (Figures 4A,B). Cyclooctasiloxane, phthalic acid, benzeneacetic acid, 9-octadecenoic acid (Z)-, methyl ester, benzoic acid, 4 methyl 2 trimethylsilyloxy, 1,2-bis(trimethylsilyl)benzene, and (4 chloro 3 nitrophenyl) methanol dimethylpentafluorophenylsilyl ether were among the specific compounds that were found. The crude secondary metabolite profiles from other treatments, including untreated controls, showed similar compositions with no discernible differences (Figure 3A; Supplementary Figure 2). Table 4 and Supplementary Table 2 offer a comprehensive list of common and induced chemicals found by GC–MS in both the cultures treated with 250 nM (UNC1999) and the untreated cultures (Ramadan et al., 2024; Yadav and Vaidya, 2024; Lutfia et al., 2021; Singh et al., 2021; Ajayi et al., 2011; Kadri et al., 2011).

**Table 4:** Induced compound in *X. papulis* culture treated with 250 nM UNC1999 identified by GC–MS analysis.

RT	Compound name	Molecular formula	M.W.	Activity	References
12.73	Cyclooctasiloxane, hexadecamethyl	C_16_H_48_O_8_Si_8_	593.2	Antimicrobial	Rasyid and Putra (2023) and Mebude and Adeniyi (2017)
15.464	Phthalic acid, hexyl propyl ester	C_17_H_24_O_4_	292.4	Toksisitas oral	Wulandari et al. (2024)
16.92	Benzeneacetic acid,?,3,4-tris[(trimethylsilyl)oxy]-, trimethylsilyl	C_20_H_40_O_5_Si_4_	472.9	-	-
20.189	9-Octadecenoic acid (Z)-, methyl ester	C_19_H_36_O_2_	296.5	Anti-inflammatory, antiandrogenic cancer preventive, dermatitigenic hypocholesterolemic, 5-alpha reductase inhibitor, anemiagenic, insectifuge	Krishnamoorthy and Subramaniam (2014)
20.43	1,2-Bis(trimethylsilyl)benzene	C_12_H_22_Si_2_	222.47	Antifungal, anti-inflammatory activity, antimicrobial activity	Ali et al. (2021)

### 3.6 Identification of compounds by UHPLC-Q-TOF-MS/MS

**Figure 5:**
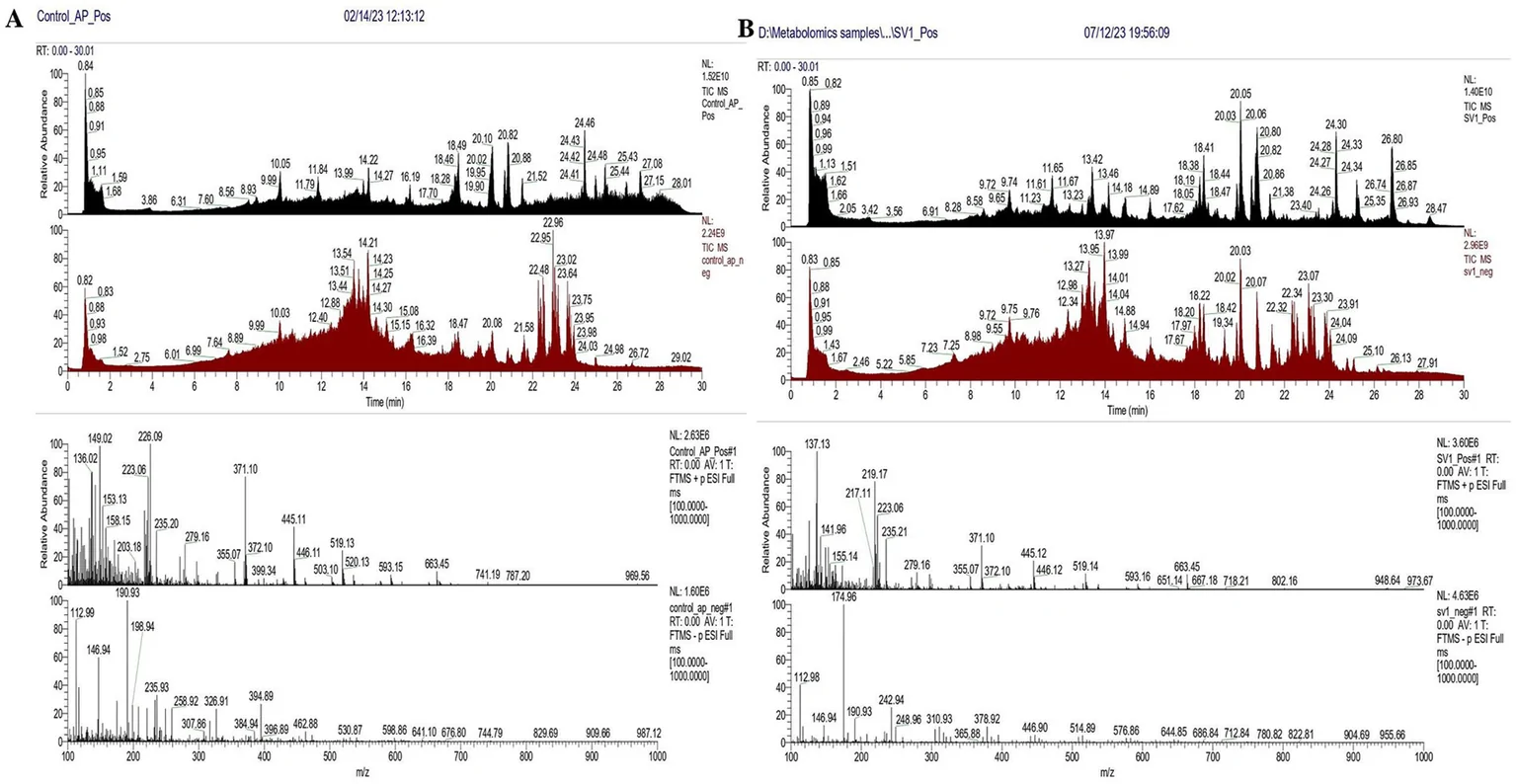
UHPLC-HRMS chromatograms of crude metabolites from **(A)** the untreated (control) sample and **(B)** the 250 nM UNC1999-treated sample, acquired in both positive and negative ionization modes.

The ultra-high-performance liquid chromatography-quadrupole time-of-flight mass spectrometry (UHPLC-Q-ToF MS/MS) technique is a powerful analytical platform for high-resolution separation, identification of unknown metabolites, and structural elucidation. In the present study, UHPLC-HRMS was employed to investigate the metabolite profiles of *X. papulis* APL1 under control conditions and after epigenetic modulation with UNC1999 (250 nM), aimed at inducing cryptic secondary metabolites (Figure 5).

Comparative UHPLC-Q-ToF MS/MS analysis demonstrated that epigenetic modification at 250 nM significantly altered the metabolic profile of *X. papulis*. Base peak chromatograms revealed notable qualitative and quantitative differences between control and treated samples. To ensure complete characterization of metabolites, mass spectrometric data were obtained in both positive and negative electrospray ionization (ESI) modes. DDA (data-dependent acquisition) was used to obtain MS/MS spectra across a mass range of *m*/*z* 50–1,500.

Accurate mass spectra determination, molecular formula prediction, and careful fragmentation pattern analysis were performed using high-resolution mass measurements. Compound identification was achieved through integration of precise mass data, elemental composition, and MS/MS fragmentation analysis. The identified metabolites, along with their chemical formulas, annotations (Delta mass, ppm), molecular weights, *m/z* values, retention times, reference ions peak areas, and group area modes, are summarized in Tables 5, 6 and Supplementary Table 3, respectively.

**Table 5:** UNC1999-treated (250 nM) induced compounds received in the positive ionization ion mode.

Metabolites	Chemical formula	Annot. DeltaMass (ppm)	Calc. MW	*m/z*	RT (min)	Area (max.)	Reference ion
Curcumin	C21H20O6	−2.16	368.12519	351.12198	17.316	10946498.6025146	[M + H-H2O] + 1
Resveratrol-4’-O-Glucuronide	C20H20O9	−2.2	404.10984	387.10663	15.157	163209407.641381	[M + H-H2O] + 1
Trans-Piceatannol	C14H12O4	−2.85	244.07286	245.08014	16.807	68236106.808424	[M + H] + 1
Caffeic acid phenethyl ester	C17H16O4	−2.3	284.1042	285.11148	16.781	22147488.4422062	[M + H] + 1
Phloretin	C15H14O5	−3.08	274.08328	275.09085	14.879	65588810.1649408	[M + H] + 1
Scopoletin	C10H8O4	−3.59	192.04157	225.07504	16.179	65633042.0040854	[M + H + MeOH] + 1
Fraxetin	C10H8O5	−3.08	208.03653	209.04382	9.774	105932567.787599	[M + H] + 1
Esculetin	C9H6O4	−3.54	178.02598	211.05946	9.872	187347029.337067	[M + H + MeOH] + 1
Vanillin	C8H8O3	−2.47	152.04697	153.05424	11.396	134199290.512731	[M + H] + 1
Vanillyl alcohol	C8H10O3	−1.62	154.06274	155.07008	15.098	9844150.47996986	[M + H] + 1
Sinapinic acid	C11H12O5	−2.66	224.06788	225.07515	15.707	173203137.549574	[M + H] + 1
Sinapyl alcohol	C11H14O4	−2.13	210.08876	211.09604	11.487	196219859.624328	[M + H] + 1
Tricin	C17H14O7	−2.94	330.07298	331.08023	15.365	51584632.305687	[M + H] + 1
Protocatechuic acid	C7H6O4	−1.97	154.02631	155.03354	4.729	51087872116.6255	[M + H] + 1
Purpurogallin	C11H8O5	−2.27	220.03667	221.04396	15.051	83268760.2636877	[M + H] + 1
Embelin	C17H26O4	−3.24	294.18216	317.17133	24.409	436396649.802932	[M + Na] + 1
Plumbagin	C11H8O3	−2.81	188.04682	189.05409	16.881	221683280.084332	[M + H] + 1
Lawsone	C10H6O3	−2.72	174.03122	175.0385	16.877	129093323.2	[M + H] + 1
Eugenol	C10H12O2	−1.32	164.08351	165.09079	15.421	94710794.7094233	[M + H] + 1
Guaiacol	C7H8O2	−1.33	124.05226	125.05958	10.51	192563301.367552	[M + H] + 1
Hydroquinone	C6H6O2	−3.23	110.03642	143.06989	11.212	188380589.289307	[M + H + MeOH] + 1
Butylated hydroxytoluene	C15H24O	−1.62	220.18236	203.17905	17.66	8215089.10027113	[M + H-H2O] + 1
TBHQ	C10H14O2	−1.78	166.09908	167.10626	17.968	477370734.426043	[M + H] + 1
Ethoxyquin	C14H19NO	−1.94	217.14624	218.15352	21.265	1360004071.76894	[M + H] + 1
e-Tokoferol	C28H42O2	−2.63	410.3174	411.32468	28.727	145826251.540754	[M + H] + 1
Urolithin B	C13H8O3	−2.24	212.04687	245.08034	17.135	122299727.31951	[M + H + MeOH] + 1
Euxanthone	C13H8O4	−2.7	228.04164	229.04892	18.716	26014586.0380067	[M + H] + 1
Xanthone	C13H8O2	−1.92	196.05205	197.05933	20.882	63618355.8448951	[M + H] + 1
Andrographolide	C20H30O5	−1.52	350.20879	392.24261	16.658	38368558.1461024	[M + ACN + H] + 1
Alantolactone	C15H20O2	−2.07	232.14585	215.14259	25.011	42524171.1231113	[M + H-H2O] + 1
Turmerone	C15H20O	−2.2	216.15094	217.1582	25.1	43512560.3770753	[M + H] + 1
Eucalyptol	C10H18O	−1.7	154.1355	137.13223	16.799	144979988.331649	[M + H-H2O] + 1
Limonene	C10H16	−1.35	136.12502	137.13229	17.27	51178454.6553873	[M + H] + 1
Trans-Cinnamaldehyde	C9H8O	−1.18	132.05736	133.06464	17.381	49254354.5578183	[M + H] + 1
Citral	C10H16O	−1.07	152.11995	305.24707	27.227	54169166.6839739	[2 M + H] + 1
Hexylresorcinol	C12H18O2	−2.29	194.13024	195.13751	20.974	56010427.2616278	[M + H] + 1
Falcarinone	C17H22O	−2.12	242.16655	243.17383	26.538	187973393.533687	[M + H] + 1
Helenalin	C15H18O4	−2.13	262.11995	263.1272	22.124	31732052.5655741	[M + H] + 1
Dehydrocostus lactone	C15H18O2	−1.6	230.13031	231.13759	25.353	6693263.05865954	[M + H] + 1
Cucurbitacin P	C30H48O7	−2.08	520.33892	521.34619	29.273	10807666.650991	[M + H] + 1
Butenafine	C23H27N	−1.85	317.21376	318.22104	28.99	106296298.498447	[M + H] + 1
Voriconazole	C16H14F3N5O	1	349.11539	350.12268	15.268	15589479.1147914	[M + H] + 1
Oxolinic acid	C13H11NO5	−1.81	261.06325	262.07053	9.277	18436655.6894295	[M + H] + 1
Novobiocic acid	C22H21NO6	−2.93	395.13573	396.14301	10.681	25178116.9128858	[M + H] + 1
Erythromycin ethylsuccinate	C43H75NO16	1.45	861.50983	884.50012	23.473	90979830.1122627	[M + Na] + 1
Oleic acid	C18H34O2	−1.2	282.25554	283.26282	30.495	9624531.34805813	[M + H] + 1
Palmitoleic acid	C16H30O2	−1.83	254.22412	255.2314	27.641	438742867.274171	[M + H] + 1
Arachidonic acid	C22H36O2	−2.29	332.27077	333.27805	29.977	3450656.44244059	[M + H] + 1
Docosahexaenoic acid	C22H32O2	−2.91	328.23927	329.24655	28.159	9544321.06798741	[M + H] + 1
Eicosapentaenoic acid	C20H30O2	−1.87	302.22402	303.23129	28.108	20410015.2766944	[M + H] + 1

**Table 6:** UNC1999-treated (250 nM) induced compounds received in the negative ionization ion mode.

Metabolites	Chemical formula	Annot. DeltaMass (ppm)	Calc. MW	*m*/*z*	RT (min)	Area (max.)	Reference ion
Secoisolariciresinol	C20H26O6	−3.24	362.17177	361.16449	23.084	5150068.6686248	[M-H]-1
Eugenol	C10H12O2	−4.6	164.08298	163.0757	8.186	13502841.3812712	[M-H]-1
Gallic acid	C7H6O5	−4.73	170.02072	169.01344	5.303	20663814.1230986	[M-H]-1
Gentisic acid	C7H6O4	−4.8	154.02587	153.0186	1.541	1938079253.83026	[M-H]-1
Scopoletin	C10H8O4	−4.89	192.04132	191.03404	25.465	13441699.729164	[M-H]-1
Morusin	C25H24O6	−2.66	420.15617	419.1489	21.618	3488186.31022764	[M-H]-1
Cyclomorusin	C25H22O6	1.85	418.14241	417.13513	8.115	471669.55489394	[M-H]-1
Schisandrin C	C22H24O6	1.78	384.15797	383.1507	17.459	9393777.53724458	[M-H]-1
Sinapyl alcohol	C11H14O4	−4.9	210.08818	210.08818	2.101	6080613.20388834	[M-H]-1
3,4-Dihydroxyphenylacetic acid	C8H8O4	−4.94	168.04143	167.03415	9.312	50108876.1758188	[M-H]-1
Dehydroascorbic acid	C6H6O6	−4.64	174.01563	173.00836	0.821	143619136.202873	[M-H]-1
Homovanillic acid	C9H10O4	−4.43	182.0571	181.04971	8.624	13762317.1169091	[M-H]-1
3,4-Dihydroxyphenylpyruvic acid	C9H8O5	−4.77	196.03624	195.02896	6.679	28407741.739725	[M-H]-1
Actinonin	C19H35N3O5	4.68	385.25947	384.2522	22.003	2132355.20144027	[M-H]-1
Thiolutin	C8H8N2O2S2	−2.76	228.00209	226.99481	8.18	7662637.51011349	[M-H]-1
Ascomycin	C43H69NO12	−4.59	791.47835	790.47107	18.621	19368614.3002535	[M-H]-1
Pyochelin	C14H16N2O3S2	2.5	324.06104	437.04663	6.604	673768.722762881	[M-H + TFA]-1

Metabolomics comparison revealed the presence of both induced and shared metabolites. Several compounds were uniquely detected in the UNC1999 with 250 nM treated samples, indicating the activation of previously silent or cryptic biosynthetic pathways, whereas some metabolites were common to both control and treated groups.

## 4 Discussion

*Andrographis paniculata* is known for its vast spectrum of pharmacological properties, which have been extracted from different plant parts (Chaturvedi et al., 2022). Leaf extracts contain anticancer, antimicrobial, and antiplatelet activities. While fruits possess higher antioxidants, anti-hyperglycemic, anticancer, and radioprotective properties, stems possess analgesic and anti-inflammatory activities (Okhuarobo et al., 2014). As this study and earlier studies showed, *A. paniculata* has a different diversity of endophytes, but the significant bioactivities obtained from the endophytes have not been reported earlier. The study identified active secondary metabolites produced from *X. papulis* APL1, which is an endophyte of *A. paniculata*, and investigated the chemical constituents (Govinda Rajulu et al., 2013; Becker and Stadler, 2021). Previous studies have shown that when fungi are treated with the proper epigenetic modifiers at the optimum dosage, cryptic or novel or targeted compound production is increased (Gupta et al., 2020; Zhang et al., 2024; Liu et al., 2025). Therefore, the isolate of *X. papulis* was grown in PDB medium with varied concentrations of UNC1999 to examine the epigenetic impact on the gene(s) responsible for producing the induced metabolites that are often not released under the normal culture conditions.

This study demonstrated a significant enhancement in antioxidant and antimicrobial activities of the fungal crude extract treated with the epigenetic modifier UNC1999 at 250 nM concentration, as indicated by a significant reduction in EC_50_ values in both DPPH and ABTS^+^ assays compared to the untreated control. This suggests that epigenetic modulation at a particular concentration significantly improved the radical scavenging potential of the extract through altered secondary metabolite production. The results of Nishad et al. (2021) corroborate the findings of this study that the treatment with histone methyltransferase inhibitor BRD4770 significantly enhances the antioxidant activity of *Diaporthe longicolla*. This enhancement is due to the epigenetic activation of gene clusters responsible for enhancing the production of antioxidant compounds such as caffeine and theobromine, which is confirmed by HPLC analysis (Nishad et al., 2021). Similarly, treating *P. heveicola* with valproic acid and 5-azacytidine showed concentration-dependent enhanced DPPH radical scavenging activity, further supporting the role of epigenetic activation in stimulating antioxidant secondary metabolism (Ameen et al., 2020). The observed improvements are strongly linked to the activation of silent BGCs, whose transcription is suppressed under normal conditions. Ramesha et al. (2021) also demonstrated that the epigenetic modifiers induce significant shifts in metabolite profiles in *Nigrospora sphaerica*, resulting in enhanced biological activities and novel compound production. The consistency between DPPH and ABTS^+^ assays in the present study further validates the robustness of the observed antioxidant enhancement, indicating broad-spectrum radical scavenging activity of the induced metabolites.

Epigenetic processes in eukaryotic organisms are highly dynamic mechanisms that lead to modulating the expression of gene(s) and are regulated through the structure of chromatin particularly regulated by methylation of DNA, phosphorylation, acetylation, noncoding RNAs, and histone post-translational modifications (PTMs) through nucleosome remodeling (Sadakierska-Chudy and Filip, 2015; Castro-Muñoz et al., 2023). The “code of histone” is made up of particular patterns of PTMs with flexible amino acids highly rich in lysine and arginine residues that are charged with N-terminus on histone tails that control compaction of chromatin and positioning of nucleosomes in facultative heterochromatin (Rutledge and Challis, 2015). PTMs are enzymes of histones that are directly involved in regulation which include PRMTs (histone-arginine N-methyltransferases) and PKMTs (histone-lysine N-methyltransferases), which are involved in catalyzing the transfer of one, two, or three methyl groups on arginine and lysine amino acids of histone proteins. The transfer of two or three methyl groups on H3K9Me2 and H3K9Me3 (lysine 9 on histone H3) catalyzed by PKMT is typically related to the repression of chromatin (Rutledge and Challis, 2015). UNC1999 acts as an inhibitor of di-methylation and tri-methylation at H3K27Me2, H3K27Me3, and PKMT. At the molecular level, these changes result in upregulation of key enzymes in secondary metabolic pathways, such as polyketide synthases (PKS), non-ribosomal peptide synthetases (NRPS), and enzymes involved in phenolic, terpenoid and flavonoid biosynthesis (Brakhage and Schroeckh, 2011). Epigenetic modifiers act as regulators of fungal metabolic plasticity, unlocking cryptic biosynthetic potential and enhancing both non-enzymatic and enzymatic antioxidant defenses, thereby reducing cellular toxicity and improving survival under oxidative stress.

Here, we observed that when treated with 250 nM of UNC1999, the crude extract of the endophytic fungus *X. papulis* showed enhanced antibacterial and antioxidative activity. The treated crude extract EC_50_ value was significantly higher than their controls. The antibacterial activity was also increased against *K. pneumoniae* (ATCC 700603), MSSA (ATCC 25923), *E. cloacae* (ATCC 13047), MRSA (MU50), and *E. coli* by their corresponding treated concentration of the UNC1999-treated crude extract. The results indicated that the production of novel active metabolites, which could not be synthesized without UNC1999 and the produced compounds could be responsible to enhanced EC_50_ for biopotential activities such as antioxidant and antibacterial activity against the above-mentioned pathogenic microbial cultures. Here, varied concentrations of UNC1999 (25 nM to 1 μM) were studied; however, only the 250 nM concentration only showed strong potential to induce cryptic compounds in broth. The GC–MS data revealed variation in the metabolite composition of treated and untreated crude samples. The GC–MS chromatogram of 250 nM UNC1999 treated crude extract clearly indicated that the non-polar compounds were induced (Table 4). The results from UHPLC-Q-TOF-MS/MS confirmed differences in the crude composition between both treated and non-treated cultures (Tables 5, 6), suggesting that certain compounds were induced only after treatment with 250 nM UNC1999. According to previous studies, the compound obtained from curcumin shows better antibacterial activity against various pathogenic bacteria, including *Enterococcus* and *Staphylococcus epidermidis* (Rai et al., 2008), and shows potent antioxidant activity (Priyadarsini, 2014). Caffeic acid phenethyl ester shows antioxidant and antibacterial properties (Taysi et al., 2023). Trans-piceatannol shows antibacterial activity against *Propionibacterium acnes*, with MICs of IC_50_ and IC_100_ of 123 and 234 mg/L (Piotrowska et al., 2012). The eugenol compound shows strong antibacterial and antioxidant activity (Marchese et al., 2017). Gallic acid compound shows strong antibacterial activity (MIC 1,500 mg/mL) (Borges et al., 2013). Plumbagin was extracted from roots of various plants, such as *Droseraceae, Plumbaginaceae*, and *Ebenaceae*. *Plumbago zeylanica* L. is known as the most efficient plant to produce plumbagin. Several studies have reported the antibacterial, antioxidant, antifungal, analgesic, anti-inflammatory and anticancer activity of this molecule (Petrocelli et al., 2023). Embelin shows antibacterial and antioxidant activity (Navalta, 2026), and scopoletin shows antioxidant and antibacterial activity (Gao et al., 2024). Esculetin is a coumarin-containing compound, which is found in various plants such as *Aesculus turbinata* and *Sonchus grandifolius*. Free radicals contribute to the development of oxidative stress (Garg et al., 2022). Protocatechuic acid has a wide range of pharmacological activities including antioxidant, antibacterial, antiviral, anticancer, antiosteoporotic, analgesia, and antiaging effects (Song et al., 2020). Tricin is known as a promising nutraceutical due to its antioxidant and anticancer properties (Ajitha et al., 2012). Purpurogallin is a phenolic compound possessing high antioxidant properties in plant-based food materials (Liao et al., 2022). Andrographolide derivatives and compounds possess antibacterial activity against MRSA by bursting reactive oxygen species (ROS) (Adiguna et al., 2023). Trans-cinnamaldehyde, citral, morusin exhibit strong antibacterial activity against MSSA ATCC 29123 (MIC 8 μg/mL), MRSA T144 (MIC 8 μg/mL), *B. subtilis* ATCC 6051 (MIC 4 μg/mL), *E. faecalis* VRE1010798 ((MIC 8 μg/mL), *E. coli* ATCC 25922 (MIC > 128 μg/mL), *E. coli* B2 (MIC >128 μg/mL), *P. aeruginosa* 14 (MIC >128 μg/mL), *K. pneumoniae* WNX-1 (MIC > 128 μg/mL), S. aureus ATCC 6538 (MIC > 6.3 μg/mL), *S. aureus* ATCC 25923 (MIC > 6.3 μg/mL), *Salmonella* ATCC 9120 (MIC >200 μg/mL), *Salmonella* DSM4224 (MIC > 250 μg/mL), *C. albicans* MIC > 60 μg/mL), *S. typhimurium* KCTC1926 (MIC > 100 μg/mL), *S. epidermidis* ATCC 12228 (MIC 20 μg/mL), and *S. aureus* (MIC 25 μg/mL) (Panek-Krzyśko and Stompor-Gorący, 2021). Secoisolariciresinol shows potent antioxidant and antibacterial properties (Smeds et al., 2007), compound which may be responsible for antioxidant and antibacterial activity identified through GC–MS and UHPLC-Q-TOF analyses.

Crude compounds from the treated culture showed antibacterial activity against *Klebsiella pneumoniae* (ATCC 700603), MSSA (ATCC 25923), *Enterobacter cloacae* (ATCC 13047), MRSA (MU50), and *E. coli*. At the same time, *Staphylococcus epidermidis* (ATCC 12223) was not affected because of the resistance genes present within plasmids of the unaffected bacterial pathogen (Rådström et al., 1991).

The treated crude extract has showed improved antioxidant activities, likely due to the presence of phthalic acid, hexyl propyl ester, benzeneacetic acid, 3,4-tris[(trimethylsilyl)oxy]-, trimethylsilyl derivative, 9-octadecenoic acid (Z)-, methyl ester induced in the GC–MS analysis, polyphenols and phenolic derivatives (curcumin, trans-piceatannol, CAPE, phloretin, scopoletin, fraxetin, esculetin, tricin, protocatechuic acid, purpurogallin, and resveratrol-4’-O-glucuronide), phenolic acids and aromatic group of compounds (vanillin, vanillyl alcohol, sinapinic acid, sinapyl alcohol, and caffeic acid derivatives), quinones and naphthoquinone group compounds (embelin, plumbagin, lawsone, and purpurogallin), phenolic volatile group compounds (eugenol, guaiacol, and hydroquinone), synthetic antioxidants (TBHQ and ethoxyquin), xanthones and its derivatives (euxanthone and xanthone), some terpenoids and sesquiterpenes (andrographolide, alantolactone, turmerone, eucalyptol, limonene, and citral), aldehydes and aromatic compounds (trans-cinnamaldehyde, citral, and hexylresorcinol), sesquiterpene lactones (helenalin, dehydrocostus lactone, and alantolactone) induced in the positive mode of UHPLC-HRMS analysis. In negative ion mode, phenolic lignans, acids and derivative compounds, such as secoisolariciresinol, gallic acid, gentisic acid, sinapyl alcohol, 3,4-dihydroxyphenylacetic acid, homovanillic acid, and 3,4-dihydroxyphenylpyruvic acid; phenolic volatile compounds (eugenol); coumarin groups (scopoletin); prenylated flavonoids (morusin, cyclomorusin); lignans (schisandrin C); and ascorbate derivatives (dehydroascorbic acid), were identified as being certainly induced due to UNC1999 treatment. Based on the findings of this study and the working principle of UNC1999 documented in previous reports, it could be suggested that a concentration of 250 nM of UNC1999 induces histone modifications after entering fungal cells, exhibiting behavior similar to that observed in mammalian cells. Ultimately, histone alteration induces upregulation or downregulation of cryptic or unknown gene(s), causing the activation and the forfeit of compounds that exist in fungal extracts. Therefore, it may be hypothesized that the activated compounds noticed in GC–MS and UHPLC-Q-TOF-MS/MS analyses may not represent all compounds induced in the treated fungus; however, it supports the effects of the epigenetic modifier UNC1999 on *X. papulis* for the production of novel or cryptic compounds.

## 5 Conclusion

This study indicates that epigenetic modification utilizing the histone methyltransferase inhibitor UNC1999 is an effective technique to unlock cryptic metabolite synthesis in the endophytic fungus *X. papulis*. The dose of 250 nM of UNC1999 used to treat cultures of the *X. papulis* was identified as an efficient concentration to trigger the recovery of bioactive cryptic metabolites, consequently boosting antibacterial and antioxidant properties. Metabolomic profiling by GC–MS and UHPLC-HRMS indicated the induction of numerous bioactive chemicals with recognized antioxidant, antibacterial, anticancer effects, suggesting that the chemical epigenetic alteration can diversify the metabolic output of fungi. This study suggests the efficacy of UNC1999 in epigenetic targets and can considerably promote the synthesis of silent compounds in the endophytic fungus *X. papulis*. The alterations in histone modification levels resulting from UNC1999 treatment may further enhance our understanding of the precise processes involved.

## Data Availability

The datasets presented in this study can be found in online repositories. The names of the repository/repositories and accession number(s) can be found in the article/Supplementary material.
