# Supplementary material for: A metabolomics approach to target the effect of histone protein methyltransferase-specific probe UNC1999 on cryptic metabolites in an endophytic fungus Xylaria papulis Lloyd
Source: Front Microbiol. 2026 Aug 14;17:1894461. doi: 10.3389/fmicb.2026.1894461 (PMC13522211; doi:10.3389/fmicb.2026.1894461)
Supplement: Supplementary file 1 [file Supplementary_file_1.docx]

**Supplementary Figure 1:** DPPH and ABTS inhibition % of non-treated and treated fungal crude extract of *X. papulis* (**A**) DPPH % inhibition of non-treated crude (**B**) DPPH % inhibition of treated crude. (**C**) ABTS % inhibition of non-treated crude (**D**) ABTS % inhibition of treated crude
