# Supplementary material for: A metabolomics approach to target the effect of histone protein methyltransferase-specific probe UNC1999 on cryptic metabolites in an endophytic fungus Xylaria papulis Lloyd
Source: Front Microbiol. 2026 Aug 14;17:1894461. doi: 10.3389/fmicb.2026.1894461 (PMC13522211; doi:10.3389/fmicb.2026.1894461)
Supplement: Supplementary file 2 [file Supplementary_file_2.docx]

**Figure 2:** GC-MS chromatogram of crude metabolites of *X. papulis* cultures treated with UNC1999 (**A**) 25 nm (**B**) 50 nm (**C**) 100 nm (**D**) 500 nm (**E**) 1000 nm
