# Supplementary material for: A metabolomics approach to target the effect of histone protein methyltransferase-specific probe UNC1999 on cryptic metabolites in an endophytic fungus Xylaria papulis Lloyd
Source: Front Microbiol. 2026 Aug 14;17:1894461. doi: 10.3389/fmicb.2026.1894461 (PMC13522211; doi:10.3389/fmicb.2026.1894461)
Supplement: Supplementary file 3 [file Table_1.DOCX]

**Supplementary Table 1**: Presents the list of taxa included in the phylogenetic analysis, along with their corresponding GenBank accession numbers, specimen numbers, geographic origin and host information. Holotype specimens are designated with the abbreviation HT, while species obtained from the present study are indicated in bold. N/A denotes information that was unavailable.

| *Xylaria papulis* | 89021903 (HAST) | Taiwan | Wood | GU300100 | GQ438744 |
| --- | --- | --- | --- | --- | --- |
| ***Xylaria papulis*** | APL1 | India | Leaf of *Andrographis paniculata* | ON796531 | PX246287 |
| *Xylaria areolata* | 543 (HAST, JF) | Guadeloupe | Bark | GU300080 | GQ408902 |
| *Xylaria tuberoides* | 475 (HAST, JF) | Martinique | Dead wood | GU300074 | GQ398232 |
| *Xylaria meliacearum* | 148 (JDR) | Puerto Rico | Petioles of *Guarea guidonia* | GU300084 | GQ408906 |
| *Xylaria hypoxylon* | NA | NA | NA | AF201711 | AY327483 |
| *Xylaria finismundoensis* | BHI-F502 | Massachusetts | Stromatal tissue | MT823483 | MT823481 |
| *Xylaria muscula* | 520 (HAST, JF) | Guadeloupe | Dead branch | GU300087 | GQ408909 |
| *Xylaria microceras* | 414 (HAST, JF) | Guadeloupe | Wood | GU300086 | GQ408908 |
| *Xylaria venustula* | 88113002 (HAST) | Taiwan | Bark | GU300091 | GQ421287 |
| *Xylaria coccophora* | 786 (HAST, JF) | French Guiana | Wood | GU300093 | GQ421289 |
| *Xylaria oligotoma* | 784 (HAST, JF) | French Guiana | Dead wood | GU300092 | GQ421288 |
| *Xylaria bambusicola* | 162 (JDR) | Thailand | Culm | GU300088 | GQ408910 |
| *Xylaria xylarioides* | GY156 | China | NA | PV608623 | PV626509 |
| *Xylaria striata* | 304 (HAST) | China | Branch | GU300089 | GQ421284 |
| *Xylaria necrophora* | DMCC2165 | USA | Root | MN846319 | MN917759 |
| *Xylaria arbuscula* | 1958 JR | USA | NA | MN846307 | MN917751 |
| *Xylaria amphithele* | 529 (HAST, JF) | Guadeloupe | Dead leaves | GU300083 | GQ408905 |
| *Xylaria cranioides* | 226 (HAST) | Taiwan | Wood | GU300075 | GQ398233 |
| *Xylaria grammica* | 479 (HAST) | Taiwan | Wood | GU300097 | GQ427197 |
| *Xylaria liquidambaris* | 93090701 (HAST) | Taiwan | Fallen fruits  of *Liquidambar formosana* | GU300094 | GQ421290 |
| *Xylaria adscendens* | 865 (JDR) | Thailand | Wood | GU322432 | GQ438746 |
| *Xylaria multiplex* | VD07152018_01 | Puerto Rico | NA | MN846338 | MN917755 |
| *Xylaria mali* | CBS 385.35 | NA | NA | KU683769 | KU684107 |
