# Supplementary material for: A metabolomics approach to target the effect of histone protein methyltransferase-specific probe UNC1999 on cryptic metabolites in an endophytic fungus Xylaria papulis Lloyd
Source: Front Microbiol. 2026 Aug 14;17:1894461. doi: 10.3389/fmicb.2026.1894461 (PMC13522211; doi:10.3389/fmicb.2026.1894461)
Supplement: Supplementary file 4 [file Table_2.DOCX]

**Supplementary Table 2:** Common compounds that were found in the fungal crude extract after treatment, identified by GC-MS analysis

| RT | Compound name | Molecular formula | M.W | Activity | References |
| --- | --- | --- | --- | --- | --- |
| 7.01 | Oleic Acid | C_18_H_34_O_2_ | 282.5 | Antimicrobial Potential | Ramadan et al., 2024 |
| 7.92 | Phenol, 2,4-bis(1,1-dimethylethyl)- | C_17_H_30_OSi | 278.5 | Antibacterial, antifungal, anticancer and Antioxidant properties | Ajai et al., 2011 |
| 10.02 | 3-Isopropoxy-1,1,1,7,7,7-hexamethyl-3,5,5-tris(trimethylsiloxy)tetrasiloxane | C_18_H_52_O_7_Si_7_ | 577.2 | Antimicrobial | Singh et al., 2021 |
| 14.96 | Cyclononasiloxane, octadecamethyl- | C_18_H_54_O_9_Si_9_ | 667.4 | Antifungal, Antioxidant | Lutfia et al., 2021, Kadri et al., 2011 |
| 20.82 | Octasiloxane, 1,1,3,3,5,5,7,7,9,9,11,11,13,13,15,15-hexadecamethyl- | C_16_H_50_O_7_Si_8_ | 578 | Antimicrobial activity | Yadavand Vaidya, 2024 |
| 30.21 | 1,2-Benzenedicarboxylic acid, butyl octyl ester | C_20_H_30_O_4_ | 334.4 | - | - |
