# Supplementary material for: A metabolomics approach to target the effect of histone protein methyltransferase-specific probe UNC1999 on cryptic metabolites in an endophytic fungus Xylaria papulis Lloyd
Source: Front Microbiol. 2026 Aug 14;17:1894461. doi: 10.3389/fmicb.2026.1894461 (PMC13522211; doi:10.3389/fmicb.2026.1894461)
Supplement: Supplementary file 5 [file Table_3.DOCX]

**Supplementary Table 3**: List of Common major compounds identified by UHPLC-HRMS analysis through negative and positive ion mode, showing chemical formula, Annot.

| **Metabolites** | **Chemical formula** | **Annot. DeltaMass [ppm]** | **Calc.**  **MW** | **m/z** | **RT**  **[min]** | **Area**  **(Max.)** | **Reference Ion** |
| --- | --- | --- | --- | --- | --- | --- | --- |
| Homovanillic acid | C9H10O4 | 0.82 | 182.0571 | 181.05078 | 3.02 | 3947694.62059985 | [M-H]-1 |
| Butylparaben | C11H14 O3 | 2.77 | 194.09394 | 193.08755 | 9.927 | 133885.381659844 | [M-H]-1 |
| S-[(1Z)-N-Hydroxy-7-(methylsulfanyl)heptanimidoyl]-L-cysteine | C11H22 N2O3S2 | -2.24, | 294.10844 | 312.1402 | 10.589 | 43651214.0863353 | [M+NH4]+1 |
| 2-Aminoadenosine | C10H14 N6O4 | -3.6, | 282.10832 | 305.09576 | 11.005 | 92817723.9997822 | [M+Na]+1 |
| 1-(6-Amino-9H-purin-9-yl)-1-deoxy-Î²-D-ribofuranuronamide | C10H12 N6O4 | -3.62 | 280.09309 | 281.09827 | 11.706 | 1161448450.3273 | [M+H]+1 |
| Pentamidine | C19H24 N4O2 | 3.93, | 340.19055 | 339.18393 | 15.357 | 3224231.65802752 | [M-H]-1 |
| Azanidazole | C10H10 N6O2 | -1.51, | 246.08763 | 247.09343 | 16.732 | 47752109.0653541 | [M+H]+1 |
| DL-Mevalonic acid | C6H12O4 | 2.11, | 148.07286 | 355.16107 | 17.083 | 21306798.2739184 | [2M-H+HAc]-1 |
| 2-Methylaminoadenosine | C11H16N6O4 | -2.78 | 296.12425 | 297.1297 | 17.276 | 33142482.3797827 | [M+H]+1 |
| Lorbamate | C12H22N2O4 | 3.5 | 258.1575 | 259.16614 | 21.213 | 18871711.2864773 | [M+H]+1 |
| Sorbic acid | C6H8O2 | 4.61 | 112.05221 | 111.04567 | 21.938 | 1589600.74417402 | [M-H]-1 |
| Tampramine | C23H24N4 | 4.51 | 356.1996 | 355.19443 | 22.057 | 903771.559554122 | [M-H]-1 |
| Tetrahydrocortisol | C21H34O5 | 1.47 | 366.23952 | 365.23389 | 22.216 | 796476.893941161 | [M-H]-1 |
| Mitoxantrone | C22H28N4O6 | -3.37 | 444.20182 | 445.20667 | 23.303 | 327167210.824907 | [M+H]+1 |
| 3beta-hydroxy-4beta-methyl-5alpha-cholest-7-ene-4alpha-carboxylic acid | C29H48O3 | -0.37 | 444.35981 | 443.35291 | 23.489 | 571306.323447633 | [M-H]-1 |
| Eliglustat | C23H36 N2O4 | -1.37 | 404.26915 | 405.27423 | 25.279 | 8113765.73450461 | [M+H]+1 |
| 5-(5-(4-(4,5-dihydro-2-oxazoly)phenoxy)pentyl)-3-methyl osoxazole | C18H22 N2O3 | 0.68 | 314.16249 | 315.17053 | 25.897 | 9425224.90883736 | [M+H]+1 |
